# Ovarian mass - tuberculosis or malignancy? Need for early intensified evaluation

**DOI:** 10.17179/excli2018-1640

**Published:** 2018-09-14

**Authors:** Shilpa N Naik, Shreya Oswal

**Affiliations:** 1 Department of Obstetrics and Gynecology, Byramjee Jeejeebhoy Government Medical College and Sassoon General Hospitals, Jay Prakash Narayan Road, Pune-01, India

## ⁯

Dear Editor, 

We read with interest the case report by Chien et al. on Peritoneal Tuberculosis (TB) mimicking ovarian cancer (Chien et al., 2016[1]). We want to congratulate the authors on this case report. 

We encountered a similar case in our clinical setting in western India. A 30-year old woman presented with intermittent fever, abdominal pain, abdominal distension and weight loss for three months. The clinical examination showed doughy feel of the abdomen and the pelvic exam showed bilateral adnexal masses. The ultrasonography showed bilateral solid cystic ovarian masses. Further, the computed tomography of abdomen and pelvis showed ascites, ovarian masses with peritoneal deposits. With these findings, the differential diagnosis of TB and ovarian cancer was considered. Laboratory investigations revealed elevated CA-125 levels (1083 U/ml). But the other tumor markers such as Alfa Fetoprotein, Lactate Dehydrogenase, Carcinoembryonic Antigen, β Human Chorionic Gonadotropin were found to be negative. Ascitic tap revealed positive Acid Fast Bacilli (AFB) and elevated ADA levels (54.4 IU/ml) that confirmed the TB diagnosis (Riqueime et al., 2006[2]). Anti-tuberculosis treatment was initiated and dramatic resolution of masses was observed on clinical examination as well as ultrasound, and CA-125 decreased to normal levels at 3 months and weight gain of over ten kilograms was seen at 6 months of TB treatment.

While a strong suspicion of ovarian malignancy that led to surgical exploration and resection of ovary was appropriate for the 56 year old case reported by Chien et al, our intensified approach towards the diagnosis of TB proved to be beneficial to the patient. The similarities between the two cases include clinical presentation and imaging findings while the investigations differed. We focused and diagnosed TB preoperatively, however surgical intervention was done in the published case and TB was diagnosed post-operatively. Such cases pose a diagnostic challenge, especially in young females.

These two cases highlight the similarities of clinical presentation between abdominal TB and ovarian malignancy. In high TB burden settings such as India, specifically for younger age groups, a full consideration of ovarian/ abdominal TB as one of the causes of ovarian masses should be given, before subjecting them to surgical interventions that include resection of ovaries, in order to avert the morbidity and induction of surgical menopause and its sequelae.

## Acknowledgement

The authors thank the study participant. This work was supported by the Obstetrics and Gynecology Department, Byramjee Jeejeebhoy Government Medical College and Sassoon General Hospitals, Pune. We would like to thank Dr. Ajay Chandanwale, Dean, BJGMC and JHU-BJGMC Fogarty Leadership. Additional support was provided by the Fogarty International Center, NIH grant number D43TW009574 (PI: Dr. Robert Bollinger). The content in this letter is solely the responsibility of the authors and does not necessarily represent the official views of the National Institutes of Health.

## References

[1] Chien JCW, Fang C-L, Chan WP (2016). Peritoneal tuberculosis with elevated CA-125 mimicking ovarian cancer with carcinomatosis peritonei: crucial CT findings. EXCLI J.

[2] Riqueime A, Calvo M, Salech F, Valderrama S, Pattillo A, Arellano M (2006). Value of adenosine deaminase (ADA) in ascetic fluid for the diagnosis of tuberculous peritonitis: a meta-analysis. J Clin Gastroenterol.

